# Effect of Plyometric Training on Vertical Jump Height in Pre‐Peak Height Velocity Boys and Girls Aged 9–11 Years

**DOI:** 10.1002/ejsc.70159

**Published:** 2026-03-28

**Authors:** Lee D. McGarrigal, Liangzhu Feng, Georgina K. Stebbings

**Affiliations:** ^1^ Department of Sport and Exercise Sciences Manchester Metropolitan Institute of Sport Manchester Metropolitan University Manchester UK; ^2^ School of Physical Education and Sports Science South China Normal University Guangzhou China

**Keywords:** boys, countermovement jump, girls, plyometric, pre‐PHV

## Abstract

This study aimed to compare changes in countermovement jump (CMJ) height and take‐off velocity, force and power in pre‐peak height velocity (pre‐PHV) boys and girls aged 9–11 years following 6 weeks of lower body plyometric training (PT). Forty pre‐PHV children (20 boys and 20 girls) were allocated to a plyometric training group (PG; *n* = 20) or a control group (CG; *n* = 20). The PG completed a progressive plyometric training programme twice weekly for 6 weeks, whereas the CG maintained usual physical activity. CMJ height and kinetic variables were assessed pre‐ and post‐intervention, and training effects were analysed using repeated‐measures ANOVA. Results demonstrated a significant between‐group difference in CMJ height (*p* = 0.018) driven by an increase in PG (5.8%; *p* = 0.013) and no change in CG (–0.5%; *p* = 0.083). No significant sex differences were observed within PG (*p* ≥ 0.359); however, CMJ height increased significantly in girls (7.0%; *p* = 0.030) but not boys (4.7%; *p* = 0.211). For kinetic variables, only take‐off velocity increased in PG (3.2%; *p* = 0.002), with comparable responses in boys (2.8%; *p* = 0.038) and girls (3.5%; *p* = 0.029), whereas force and power showed no significant changes (*p* ≥ 0.202). No significant changes were observed in CG for CMJ or kinetic variables (*p* ≥ 0.083). In conclusion, plyometric training improved CMJ performance in pre‐PHV children, with no significant sex differences observed. Training adaptations at this stage should be interpreted considering biological maturation status.

## Highlights

- A 6‐week, twice a week, progressive lower body plyometric training programme significantly increased countermovement jump (CMJ) height in the plyometric group.
- Pre‐PHV girls significantly increased CMJ height, but aged‐matched boys did not.
- Plyometric training increased take‐off velocity speed, but not force or power.
- It appears that pre‐PHV girls experienced synergistic adaptation following plyometric training, whereas pre‐PHV boys did not.
- Age and maturity status impacts the positive effect of plyometric training.

## 1 Introduction

Jumping is an essential component of many sports (Asadi et al. 2016; Darmiento et al. 2012; Emmonds et al. 2018; McGarrigal et al. 2025b), and therefore is commonly included in battery tests used by strength and conditioning practitioners (Faude et al. 2012; J. M. Taylor et al. 2022). Due to relative simplicity (Bishop et al. 2022), time efficiency (Bishop et al. 2017), validity and reliability (Markovic et al. 2004), countermovement jump (CMJ) height is often used to evaluate the lower‐body power and strength of athletes and children (Emmonds et al. 2018; Markovic et al. 2004; Moran et al. 2018; Slimani et al. 2017). A CMJ is often associated with 0–30m sprinting and change of direction performance (Markström and Olsson 2013; Plesa et al. 2022) and can also be used to assess neuromuscular fatigue, the effectiveness of training, readiness to train, and to predict injury risk (Bishop et al. 2023; Plesa et al. 2022). Collectively, this makes a CMJ a useful tool for strength and conditioning practitioners aiming to monitor the physical fitness and lower‐body power of their athletes. The CMJ also assesses the slow (> 250 ms) stretch‐shortening cycle (SSC) (R. S. Lloyd, Oliver, et al. 2011; Van Hooren and Zolotarjova 2017), which includes an eccentric contraction, quickly followed by a concentric contraction (Turner and Jeffreys 2010). Due to the inclusion of an eccentric phase, force production is increased beyond that of a concentric‐only jump, such as a squat jump (Bobbert and Casius 2005; Bobbert et al. 1996).

One popular method often used to enhance CMJ height is plyometric training (PT) (Markovic 2007; Markovic and Mikulic 2010; Moran et al. 2018). Commonly, PT incorporates skipping, hopping, jumping and bounding exercises to use several neurophysiological and mechanical mechanisms of the SSC (Potach and Chu 2016; Turner and Jeffreys 2010). Although it is well‐known that PT increases the physical performance of children (Asadi et al. 2018; Asadi et al. 2016; Moran et al. 2018; Ramirez‐Campillo, Gallardo, et al. 2015), age and maturation may affect the optimisation of PT. For example, Moran et al. 2018 reported that girls aged < 15 years increased jump height more than girls aged > 15 years. Additionally, research studies show that pre‐peak height velocity (PHV) boys generate greater training adaptations using PT‐only than post‐PHV boys, who gain a greater response from combined PT and resistance training (R. S. Lloyd, Radnor, et al. 2015). This result may reflect the fact that the heightened neural plasticity and increased sensitivity for motor control and coordinative adaptation in pre‐PHV children improve to a greater degree due to the high neural demands of PT (Myer et al. 2015, 2013), whereas post‐PHV boys generate greater SSC improvements by experiencing an enhanced hormonal profile and hypertrophic adaptation associated with adolescence (Faigenbaum et al. 2013; R. S. Lloyd, Radnor, et al. 2015). Such findings have been termed ‘synergistic adaptation’, which refers to a symbiotic relationship between a training stimulus and the increase in SSC function due to growth and maturation. Although fewer studies have examined maturation‐related adaptations to PT in girls compared with boys, synergistic adaptation has recently been observed in youth female soccer players during maturation (McGarrigal et al. 2025b). In that study, several measures of SSC function, including CMJ height, increased to a similar degree across pre‐, mid‐ and post‐PHV girls following PT.

Prior to the physiological, hormonal and anatomical changes that occur during maturity, boys and girls commonly record similar jump heights (Malina et al. 2004a). Following PHV, although boys jump higher (Malina et al. 2004a), both sexes may also adapt equally well to PT (Ramirez‐Campillo et al. 2023). However, there is currently a lack of research studies comparing the PT response of pre‐PHV boys and girls following the same programme (Faigenbaum et al. 2009; Steben and Steben 1981). Finding that pre‐PHV boys and girls adapt differently in CMJ performance following PT could influence when strength and conditioning practitioners introduce this method of training in youth‐based settings. Therefore, this study aimed to compare the training response in pre‐PHV boys and girls aged 9–11 years in vertical jump height following the same 6‐week, twice per week PT. It was hypothesised that plyometric training would improve CMJ performance in both boys and girls, with no significant sex differences in training adaptations.

## 2 Method

### 2.1 Participants

Forty pre‐PHV children (aged: 10.5 ± 0.5 years; maturity offset: −2.3 ± 0.9 years; height: 1.4 ± 0.1 m; mass: 38.0 ± 9.3 kg; body mass index [BMI]: 18.1 ± 3.4 kg·m^−2^; leg length: 0.5 ± 0.9 m; Table 1) volunteered to take part in this study. Using blinded randomized allocation (Sammoud et al. 2022), 20 girls and 20 boys were split evenly between the PT intervention group (PT; *n* = 20; 10 girls and 10 boys; Table 1) and the control group (CG; *n* = 20; 10 girls and 10 boys; Table 1). As all participants were under 18 years, parents or guardians signed a consent form and completed a pre‐activity questionnaire (PARQ) on behalf of their child. Any child with a preexisting injury was withdrawn from the study. All procedures complied with the last Declaration of Helsinki (World Medical Association, 2013). All children participated in 2‐one hour phyiscal education lessons per week (i.e., tennis) and all participants had experience of performing organised PT.

**TABLE 1:** Anthropometric characteristics of participants by group and sex at baseline and post‐training. Data are presented as mean ± SD and *p*‐values.

	Control group (*n* = 9)		Plyometric group (*n* = 21)	
	Baseline	Post‐training	Baseline	Post‐training
Age (yrs.)				
Boys	10.3 ± 0.3	10.4 ± 0.3	10.2 ± 0.5	10.3 ± 0.6
Girls	10.4 ± 0.1	10.6 ± 0.2	10.7 ± 0.6*	10.8 ± 0.8*
Combined	10.3 ± 0.2	10.5 ± 0.3	10.4 ± 0.6	10.6 ± 0.7
PHV offset (yrs.)				
Boys	−3.0 ± 0.5	−2.9 ± 0.8	−3.0 ± 0.5	−3.0 ± 0.5
Girls	−1.8 ± 0.3*	−1.7 ± 0.6*	−1.4 ± 0.2^a^	−1.3 ± 0.8^a^
Combined	−2.4 ± 0.9	−2.3 ± 0.9	−2.3 ± 1.1	−2.2 ± 1.1
Height (m)				
Boys	1.5 ± 0.1	1.5 ± 0.1	1.4 ± 0.1	1.5 ± 0.1
Girls	1.4 ± 0.1	1.4 ± 0.1	1.4 ± 0.1	1.4 ± 0.1
Combined	1.4 ± 0.1	1.4 ± 0.1	1.4 ± 0.1	1.5 ± 0.1
Body Mass (kg)				
Boys	42.4 ± 14.5	40.2 ± 15.3	39.8 ± 10.8	40.2 ± 10.7
Girls	34.5 ± 9.5	34.1 ± 9.1	34.4 ± 7.4	34.5 ± 7.5
Combined	38.9 ± 12.5	37.5 ± 12.6	37.2 ± 9.5	37.4 ± 9.6
BMI (kg·m^−2^)				
Boys	19.3 ± 4.3	18.3 ± 5.1	19.1 ± 4.2	19.0 ± 4.2
Girls	17.4 ± 3.6	17.1 ± 3.3	16.4 ± 1.9	16.4 ± 1.9
Combined	18.4 ± 3.9	17.8 ± 4.2	17.9 ± 3.5	17.7 ± 3.5
Leg length (m)				
Boys	0.7 ± 5.4	0.7 ± 5.4	0.7 ± 1.1	0.7 ± 1.1
Girls	0.7 ± 6.2	0.7 ± 6.2	0.7 ± 1.1	0.7 ± 1.1
Combined	0.7 ± 5.7	0.7 ± 5.7	0.7 ± 4.5	0.7 ± 4.5

Abbreviation: PHV, Peak height velocity.

^a^ *p* < 0.01 Significant difference between boys and girls.

^*^ *p* < 0.05 Significant between boys and girls.

### 2.2 Anthropormetric Measures

Standing and sitting height were measured to the nearest 0.1 cm with the use of a free‐standing stadiometer (Seca 213 stadiometer, Seca GmbH, Hamburg, Germany), with participants measured barefoot. Sitting height was measured using the stadiometer, with the participants sitting up straight in a chair, with their feet flat on the floor (Franklin et al. 2024). Leg length was calculated by subtracting the sitting height from the standing height (Franklin et al. 2024). Body mass was measured to the nearest 0.1 kg using calibrated digital scales (Seca 813, Seca GmbH, Hamburg, Germany) with participants barefoot and wearing light clothing (shorts and a t‐shirt). Body Mass Index (BMI) was calculated as body mass in kg divided by the square of height in metres (Sharma et al. 2017). All anthropometric measures are displayed in Table 1.

### 2.3 Maturity Offset

Predicted years from PHV was measured according to Mirwald et al. 2002 using sex‐specific equations for girls (Equation 1) and boys (Equation 2), with pre‐PHV categorised as being < −1 year away from PHV (McGarrigal et al. 2025b). Maturity offset is displayed in Table 1.

(1) Femaleoffset=−9.376+(0.0001882·(leglength·sittingheight))−(0.0022·(age·leglength))+(0.005841·(age·sittingheight))−(0.002658·(age·bodymass))+(0.07693·(bodymass÷stature))·100

(2) Maleoffset=−9.236+(0.0002708·(leglength·sittingheight))+(−0.001663·(age·leglength))+(0.007216·(age·sittingheight))−(0.02292·(bodymass÷stature))·100

where stature, leg length and sitting height is measured in cm, body mass is measured in kg and age is chronological age in years and months at the time of testing (Mirwald et al. 2002).

### 2.4 Familarisation

Prior to testing, participants attended two familiarisation sessions over 2 weeks, separated by 7 days. During these sessions, the lead researcher (who has experience of testing, performing and observing jump tests in youth populations) demonstrated and coached participants how to correctly perform a CMJ used during testing. To standardise CMJ height, the lead researcher observed each participant and made sure the performance of CMJ was satisfactory (e.g., in accordance with the guidelines of procedure) and until no further improvements in performance were observed (e.g., no increase in jump height). The last CMJ of each participant was then pooled and used to test absolute agreement and absolute reliability of CMJ height (R. S. Lloyd et al. 2009). Motivation during each jump was maintained through verbal encouragement and competition between athletes (Flanagan and Comyns 2008). All familiarisation and testing sessions included a 5‐min warm up comprising bodyweight squats, heel flicks, high knees and repeated CMJs in place for 2 sets of 10 repetitions each. Participants were asked to wear the same clothing and footwear throughout this study, and to avoid drinking, eating and exercising 1 hour before each familiarisation and testing session (R. S. Lloyd et al. 2009). To minimize the influence of external factors (e.g., weather, surface interaction, and interrater inconsistences), all tests were completed at same temperature‐controlled indoor venue by the lead researcher, on a hard wooden surface (Ramirez‐Campillo, Meylan, et al. 2015).

### 2.5 Instrumentation

Traditionally, vertical jump height has been tested in a laboratory‐based setting using ‘gold standard’ force platforms (Linthorne 2001). However, due to their inaccessibility, price and lack of transportability of force platforms, strength and conditioning practitioners often seek more cost‐effective field‐based devices to test the jump height of athletes (McMahon et al. 2019). In the current study, the valid and reliable MyJump 2 app (Apple Inc. Cupertino, USA) was used to measure CMJ height (Gençoğlu et al. 2023; Sharp et al. 2019). In accordance with the designers, the lead researcher lay prone 1.5 m away from each participant and filmed their landing and take‐off time (Balsalobre‐Fernandez et al. 2015) using the slow‐motion capabilities of an Apple 12 iPhone (Apple Inc., Cupertino, USA). In accordance with the app's instructions, body mass (kg), leg length (cm) and squatting height (cm) was entered into the MyJump 2 software prior to testing.

### 2.6 Pre and Post Testing

Each participant performed a pre‐ and post‐testing session during Week 1 and Week 6, respectively. To minimize the positive effect of arm swing, all CMJs involved the participants standing with their hands on their hips throughout the movement (Floria and Harrison 2013; Gerodimos et al. 2008) and then lowering themselves from an initial standing position to a self‐selected squat position, followed immediately by a vertical jump (R. S. Lloyd et al. 2009). Participants were encouraged to perform the eccentric phase (downward) of the jump as quickly as possible, with maximum effort, to maximize jump height (Cormack et al. 2008). Motivation during each jump was maintained through verbal encouragement and competition between athletes (Flanagan and Comyns 2008). All participants performed the CMJ thrice, with the best jump height used for further analysis (R. S. Lloyd et al. 2009).

### 2.7 Plyometric Training

PT was conducted over 6 weeks and performed twice per week, with sessions separated by 72 hours (Table 2). Each session commenced with standardized warm‐up consisting of light jogging, jumping jacks and high knee drills in place for 5 min. PT was progressed over the course of the 6‐week intervention by increasing volume and intensity (R. Lloyd et al. 2011). Training volume was defined by the number of foot contacts made during each session (Ebben et al. 2010), with a contact identified each time the lower extremities perform one attempt of each exercise. Progressive volume‐based PT was used because it appears more advantageous that non‐progressive PT (Ramirez‐Campillo et al. 2020). The PT programme started with 80 contacts in each session of week 1, increasing by 20 jumps per session (weeks 1–5), up to a total of 160 contacts in each session in week 5, before tapering to 60 contacts per session in week 6 (Ramirez‐Campillo et al. 2020). Specifically, Ramirez‐Campillo et al. (Ramirez‐Campillo et al. 2020) reported that *a* > 40% reduction in volume has been shown to have a greater effect size (ES) on jump capacity than tapering volume ≤ 40% (effect size [ES] = 1.18 and 0.61, respectively). Plyometric drills lasted approximately 5–10 s, with 60–90 s rest between sets of the same exercises and 2 min rest between different tasks (Meylan and Malatesta 2009; Moran et al. 2024). This work–rest ratio was enforced to optimise repetition velocity, enable full‐recovery and probably retain maximal motor unit recruitment throughout the training session (R. S. Lloyd, Oliver, et al. 2012). To increase elastic energy reutilization and excite the stretch reflex (R. S. Lloyd et al. 2009), all plyometric exercises were performed continuously (no pause between reps) (Moran et al. 2024). Owing to a lack of PT familiarity, verbal feedback focused on correct take‐off and landing mechanics (R. S. Lloyd, Oliver, et al. 2012). The training intervention was supervised by the lead researcher who was experienced strength and conditioning practitioner experienced in performing and coaching PT (R. Lloyd et al. 2011). Exercise quality was carefully observed to ensure proper execution and limit the risk of injury (Sylvester et al. 2024), with no injuries occurring during the intervention.

**TABLE 2:** 6‐Week plyometric training programme.

Exercises^a^ ^,^ ^b^	Week 1	Week 2	Week 3	Week 4	Week 5	Week 6
Pogo hops	2 × 20	2 × 10	2 × 10	3 × 10	2 × 20	
Countermovement jumps	2 × 10	2 × 10	2 × 10	3 × 10	2 × 10	2 × 10
Tuck jumps		2 × 10	2 × 10	2 × 10	2 × 10	2 × 10
Jump and reach		2 × 10	2 × 10	2 × 10	2 × 10	2 × 10
Vertical bilateral hopping	2 × 10	2 × 10	2 × 10	2 × 10	2 × 10	
Vertical unilateral hopping			2 × 10	2 × 10	2 × 10	
Total foot contacts per leg	80^c^	100^c^	120^c^	140^c^	160^c^	60^c^

*Note:* Intensity: exercises were performed with maximal effort (intensity: 100%), Rest: 60–90 s min between sets of same exercise. Exercises (all continuous jumps with no pause between reps, with 2 min rest between sets of different exercises) (Moran et al. 2024).

^a^ The order of exercise execution was randomized each session.

^b^ All exercises were executed with the technique described as countermovement without arms.

^c^ The total number of foot contacts described as per session, which remained that same for 2 plyometric sessions completed each week. Volume increased by 20 jumps per session in week one to five, before tapering off in week 6 (Sammoud et al. 2022).

In accordance with the principle of training specificity (Behm and Sale 1993), all plyometric exercises were performed in the vertical plane (Ramirez‐Campillo, Gallardo, et al. 2015). To minimize the positive effect of arm swing, all plyometric exercises were performed with arms akimbo (Floria and Harrison 2013; Gerodimos et al. 2008). To maintain motivation and variety, the order of exercises was randomized for each training session (Ramirez‐Campillo, Gallardo, et al. 2015). All groups performed PT on the same hard surface throughout all testing and intervention sessions. Due to a lack of prior plyometric experience, PT used a tester‐to‐participant ratio of 1:5 (Potach and Chu 2016), with attention paid to demonstration, correct execution, verbal feedback, focus on correcting posture, linearity of jumping kinematics and take‐off and control of landing mechanics (Cronin and Radnor 2020).

### 2.8 Statistical Analysis

The sample size estimation was computed using G*Power software (version 3.1.9.4) (Faul et al. 2009). With reference to a previous study on the effect of 12 weeks PT on maximal strength performance (i.e., 1RM half‐squat), linear sprinting and change of direction speed in pre‐PHV male soccer players, a priori power analysis, with a type I error rate of 0.05% and 80% statistical power was computed (Sammoud et al. 2022). A total of 10 participants per group would yield a power of 80% and *α* = 0.05 (Sammoud et al. 2022). A two‐way mixed effect, absolute agreement and intraclass correlation coefficient (ICC), with 95% confidence intervals (CI) was used to examine the test‐retest reliability for CMJ height across two familiarisation sessions using the MyJump 2 app. Based on previous literature, ICC was classified as poor (< 0.50), moderate (0.50–0.74), good (0.75–0.89) and excellent (> 0.90) (Koo and Li 2016). Absolute reliability using 95% CI coefficient of variance (CV) was classified as poor (≥ 15%), moderate (10%–15%), good (5%–10%) and excellent (≤ 5%) (Franklin et al. 2024).

To analyse group differences in anthropometric variables and any changes between PRE‐POST performance, Statistics Package for Social Sciences (SPSS) version 28 (IBM Corporation, New York, USA) was used. Assumptions of parametricity were checked using Shapiro‐Wilk to assess normality and Levene's test of homogeneity of variance. Two‐way analysis of variance (ANOVA) with repeated measures (2 times × 2 groups) and (2 times × 2 sex) using Bonferroni post‐hoc was used to determine the effects of plyometric training on the dependent variables. Changes in dependent variable performance from baseline‐to post‐training are represent by % difference. One‐way ANOVA was used to calculate baseline‐to post‐training CMJ height and its associated determinants of take‐off velocity, force, force relative to body mass, power and power relative to body mass, with 95% confidence intervals. Effect size (ES) was determined using the modified Cohen's *d* scale, classified as trivial (≤ 0.2), small (0.2–0.6), moderate (0.6–1.2), large (1.2–2.0), very large (2.0–4.0), and extremely large (≥ 4.0) (Hopkins 2002). Statistical significance level was set at *p* < 0.05 (Moeskops et al. 2018), and data are presented as mean ± standard deviation (SD).

## 3 Results

Test‐retest CMJ absolute agreement and absolute reliability between the final jumps between each familiarisation session was ‘excellent’ based on ICC = 0.966 (0.934–0.986; ES = 0.02) and CV = 3.7% (2.8%–4.5%). There were no differences between CG and PG, or between PG boys and PG girls for any anthropometric variables at baseline or post‐training (*p* ≥ 0.089; Table 1). There was also no significant difference in age and predicted yrs. from PHV between CG and PG (Table 1). There was, however, a significant difference in the age of PG boys and PG girls (Table 1), in predicted yrs. from PHV of CG boys and CG girls (*p* ≤ 0.039), and between PG boys and PG girls (*p* ≤ 0.001) at both time points.

Following 6 weeks of plyometric training, CMJ height was significantly greater in the plyometric group (PG) compared with the control group (CG) (*p* = 0.018; Table 3), with CMJ height significantly increasing the PG (Table 3), with no change in the CG (Table 3). There was no significant difference in CMJ height between PG boys and PG girls prior to or following the intervention (*p* ≥ 0.359). However, the PG girls demonstrated a significant increase in CMJ height (Table 3), whereas PG boys did not (Table 3). Prior to training, the PG demonstrated significantly greater CMJ height, power, force, power and force‐to‐weight ratio (*p* > 0.001; Table 4), which was not evident post‐training (*p* ≥ 0.137; Table 4). Take‐off velocity increased significantly in PG, PG boys and PG girls (Table 4). No significant changes were observed in jump force, jump power, power and force‐to‐weight ratio in any group after 6 weeks.

**TABLE 3:** Baseline and post‐training for CMJ height, percentage change (95% CI), *p*‐values (95% CI), and modified Cohen's *d* (ES, 95% CI). Data are presented as mean ± standard deviation (SD).

	Baseline Mean ± SD	Post‐training Mean ± SD	Performance change % (95% CI)	*p*‐value	ES (95% CI)
CMJ (cm)					
Control	21.6 ± 2.8	21.5 ± 3.2	−0.5 (−5.9 to 0.4)	0.083	−0.04 (−0.18 to 0.09)
Plyometric	22.2 ± 3.8	23.5 ± 4.6^a^	5.8 (1.6–10.0)	0.013*	0.31 (0.17–0.45)
PG boys	22.1 ± 3.4	22.9 ± 3.0	4.7 (−2.1–11.5)	0.211	0.26 (−0.02–0.53)
PG girls	22.3 ± 4.3	24.1 ± 5.8	7.0 (0.7–13.3)	0.03*	0.35 (0.07 to −0.63)

*Note: p*‐value is reflecting baseline and post‐training comparison; modified Cohen's d.

Abbreviations: CI, confident intervals; CMJ, countermovement jump; ES, effect size.

^a^ *p* < 0.01 Significant difference with CG post‐training.

^*^ *p* < 0.05 Significant within‐groups significance.

**TABLE 4:** Baseline and post‐training for velocity, force and power, percentage change (95% CI), *p*‐values (95% CI), and modified Cohen's *d* (ES, 95% CI). Data are presented as mean ± standard deviation (SD).

	Baseline Mean ± SD	Post‐training Mean ± SD	Performance change % (95% CI)	*p*‐value	ES (95% CI)
Velocity (ms)					
Control	1013.1 ± 94.2	1009.2 ± 83.4	−0.3 (−1.2–1.1)	0.081	−1.0 (−1.15–0.09)
Plyometric	1035.3 ± 86.4	1068.4 ± 104.3^a^	3.2 (1.3–5.1)	0.002^a^	1.0 (0.85–1.15)
PG boys	1028.1 ± 73.1	1057.0 ± 71.3	2.8 (0.1–5.9)	0.038*	1.0 (0.71–1.29)
PG girls	1041.2 ± 101.4	1078.1 ± 133.4	3.5 (0.5–6.5)	0.029*	1.0 (0.71–1.29)
Force (*N*)					
Control	662.8 ± 148.5	646.8 ± 130.3	−1.7 (−6.6–3.2)	0.328	−0.11 (−0.25–0.02)
Plyometric	655.1 ± 173.4	678.1 ± 219.7	1.7 (−5.4–8.8)	0.637	0.12 (−0.02–0.25)
PG boys	697.5 ± 198.6	689.7 ± 201.8	−0.3 (−8.6–8.0)	0.805	−0.04 (−0.32–0.24)
PG girls	629.6 ± 142.5	665.3 ± 248.4	4.0 (−9.7–17.6)	0.464	0.18 (−0.10–0.45)
Power (W)					
Control	630.2 ± 156.2	601.1 ± 126.7	−3.7 (−10.0–2.7)	0.202	−0.20 (−0.34–−0.02)
Plyometric	688.4 ± 185.5	719.0 ± 241.1	4.1 (−3.1–11.4)	0.303	0.14 (0.0–0.28)
PG boys	714.1 ± 187.6	722.4 ± 195.4	2.3 (−7.5–12.2)	0.829	0.04 (−0.23–0.32)
PG girls	660.2 ± 188.9	715.3 ± 294.4	6.1 (−6.7–18.9)	0.256	0.22 (−0.06–0.32)
N.kg^−1^					
Control	17.7 ± 3.2	18.0 ± 3.0	2.7 (−7.1–12.4)	0.664	0.10 (−0.04–0.24)
Plyometric	17.9 ± 1.7	18.0 ± 2.8	1.1 (−5.9–8.0)	0.850	0.04 (−0.10–0.18)
PG boys	17.6 ± 2.0	17.2 ± 2.0	−1.2 (−9.8 to 7.4)	0.643	−0.20 (−0.48–0.08)
PG girls	18.3 ± 1.2	18.9 ± 3.4	3.5 (−9.3–16.4)	0.548	0.24 (−0.04–0.51)
W.kg^−1^					
Control	17.1 ± 5.0	17.0 ± 4.5	0.3 (−7.3–7.9)	0.846	−0.02 (−0.16–0.12)
Plyometric	18.6 ± 2.9	19.2 ± 4.2	3.5 (−3.8–10.7)	0.390	0.17 (0.03–0.31)
PG boys	18.1 ± 2.9	18.2 ± 2.9	1.4 (−8.8–11.7)	0.972	0.03 (−0.24–0.31)
PG girls	19.1 ± 2.9	20.3 ± 5.2	5.7 (−6.5–18.0)	0.280	0.29 (0.01–0.56)

*Note: p*‐value is reflecting baseline and post‐training comparison; modified Cohen's d.

Abbreviations: CI, Confidence intervals; ES, effect size; N.kg^−1^, Force relative to body mass; W.kg^−1^, power relative to body mass.

^a^ Significant difference with CG post‐training (*p* < 0.05).

^*^ Denotes significant within‐groups significance (*p* < 0.05).

## 4 Discussion

This study aimed to compare improvements in CM height and its derivatives of take‐off velocity, force and power in pre‐PHV boys and girls aged 9–11 years following 6 weeks, twice per week, lower body PT. The study hypothesis was supported, as CMJ performance improved following PT, with no significant sex differences in training adaptations. The main findings are that CMJ height and take‐off velocity increased in the PG following PT (+5.8%, *p* = 0.013; ES = 0.31 and + 3.2%, *p* = 0.002; ES = 1.0, respectively). In addition, although there was no significant difference between the CMJ performance of boys and girls following PT (*p* ≥ 0.546, ES ≤ 0.26; Table 3), PG girls significantly increased CMJ height (+7%; *p* = 0.03; ES = 0.35), whereas PG boys did not (+4.7%; *p* = 0.211; ES = 0.26). Given the statistically significant improvements in the CMJ height of PG girls, but the none‐significant group difference based on sex, broadly similar PT adaptations between boys and girls occured aged 9–11 years following PT.

It has been stated that the SSC is governed by neural regulation (Markovic and Mikulic 2010; Radnor et al. 2018), and research studies show that pre‐PHV is a time frame during which children experience a proliferation in neural coordination, central nervous system maturation and neural plasticity (Borms 1986; Myer et al. 2013; Sowell et al. 2004) that positively responds to the high neural demands of PT (R. S. Lloyd, Radnor, et al. 2015; Markovic and Mikulic 2010; Myer et al. 2013). Therefore, it was reasonable to predict that PT would have led to an improvement in jump height in all participants (Ramirez‐Campillo et al. 2023). Positive improvements following PT may occur due to several neural adaptations (Legerlotz et al. 2016), including increased neural drive to the agonist muscles, improved intermuscular coordination, changes in musculotendinous mechanical stiffness characteristics, changes in muscle size or architecture, changes in single‐fibre mechanics (Markovic and Mikulic 2010), as well as the development of active state (Bobbert and Casius 2005; Bobbert et al. 1996), working range and impulse (Kirby et al. 2011; Ruddock and Winter 2016). However, as no physiological or biomechanical measurements were taken, these assumptions are speculative and warrant further research.

In the current study, PG girls improved CMJ height, whereas PG boys did not. However, given the absence of a significant sex interaction, these findings should be interpreted cautiously and do not necessarily indicate a true sex‐specific adaptation. Interestingly, girls may use more stored elastic energy during a CMJ than boys (90% vs. 50%) (Komi and Bosco 1978), which may mean girls have higher slow SSC efficiency than boys. Nevertheless, such explanations remain speculative and cannot be confirmed within the present study design. Importantly, this does not imply that pre‐PHV boys cannot benefit from PT alone, as suggested in previous literature, but rather that training responsiveness may vary depending on individual maturation status and programme characteristics (Behm et al. 2008; R. S. Lloyd, Radnor, et al. 2015).

The finding that girls improve SSC function more than boys disagrees with one systematic review with meta‐analysis, which reported that girls and boys adapted equally following PT (Ramirez‐Campillo et al. 2023). However, the same authors did not include studies that directly compared boys and girls using the same PT programme. The current finding, however, does concur with one meta‐analysis that found that female soccer players aged 15–21 years increased CMJ height to a greater degree than their male counterparts (*Q* = 4.52; *p* = 0.033) (Slimani et al. 2017). Nevertheless, Slimani et al. 2017 did not include pre‐PHV male and female soccer players, and so it remains unclear whether pre‐PHV girls would improve vertical jump height to a greater degree than mid‐ and post‐PHV girls following the same PT. Consequently, future studies should include more PT studies involving pre‐PHV populations, as the findings may influence when strength and conditioning practitioners include PT in training programs aimed at pre‐PHV boys and girls in a youth‐based setting.

One further reason for the girls experiencing a greater increase in CMJ height than the boys following PT might be that the girls had entered their adolescent growth spurt and their ‘window of training opportunity’, resulting an increase in power, hypertrophy, speed and strength, which the boys had yet to experience (Balyi and Hamilton 2004; R. Lloyd and Oliver 2012). This assumption is reasonable, given that boys typically enter their own adolescent growth spurts 2 years after girls (Malina et al. 2004b). Indeed, the estimated maturity status of the participants in the current study would support this, as PG girls were significantly more mature (*p* < 0.001) closer to their predicted PHV than PG boys (−1.4 ± 0.8 years vs. −3.0 ± 0.5 years) (R. S. Lloyd, Oliver, et al. 2015). This is pertinent given that ∼1‐year pre‐PHV can offer an accelerated period of SSC development, where pre‐PHV children respond favourably to short‐term PT (R. S. Lloyd, Radnor, et al. 2015). Therefore, proximity to PHV, rather than the existence of a distinct ‘window of training opportunity,’ may partly explain the observed differences in maturity. This interpretation acknowledges the complexity of training adaptations during growth and emphasises the importance of considering maturation when prescribing youth training.

The current results also demonstrated a significant increase take‐off velocity in the PG (*p* = 0.002; ES = 1.0), but not in force and power (*p* ≥ 0.303; ES ≤ 0.12), or in force and power in relation to body mass (*p* ≥ 0.390; ES ≤ 0.17). Additionally, unlike CMJ height, take‐off velocity significantly increased in both the PG girls and boys following PT (*p* ≤ 0.038; ES = .1.0). It is well‐established that vertical jump height is positively correlated with execution velocity (Badillo 2017; Gonzalez‐Badillo and Marques 2010). However, although take‐off velocity improved in both sexes, this did not translate into statistically significant improvements in CMJ height in boys or meaningful changes in power output. Jump height is influenced not only by take‐off velocity but also by impulse, movement strategy and the consistency of force application. Relatively small improvements in velocity (≈3%) may not have been sufficient to overcome inter‐individual variability and produce statistically significant increases in overall jump performance in boys. Furthermore, the absence of changes in force and power suggests that improvements in velocity may have reflected enhanced coordination or timing of force production rather than increases in maximal force capacity. Such neuromuscular refinements may improve specific performance variables without substantially altering global power measures.

As CMJ height in girls significantly increased, it is possible that following PT, girls performed the CMJ with a greater eccentric depth in the countermovement phase, which improved the active state development, impulse and reutilisation of stored elastic energy to increase jump height (Kirby et al. 2011; Potach and Chu 2016; Ruddock and Winter 2016). It might also be reasonable to speculate that the PG demonstrated improved motor coordination following PT compared to the CG to improve vertical jump height (Maarten et al. 2013; Markovic and Mikulic 2010). However, these assumptions are speculative. Therefore, to fully identify the influences on CMJ height, future research studies may wish to measure countermovement depth using video analysis and force plates to measure jump duration, time‐to‐take off, impulse, ground reaction forces, force‐time curve (Anicic et al. 2023), impact peak ground force and ‘spring‐like’ behaviour (Moeskops et al. 2020; Pedley et al. 2020). Although some literature suggests that post‐PHV individuals may benefit more from combined PT and resistance training, the present findings do not directly test this comparison. Therefore, recommendations regarding the inclusion of resistance training in pre‐PHV populations should be interpreted cautiously and tailored to the individual's maturation status, training history and performance goals.

In this study, only slow SSC function was developed following PT (R. S. Lloyd, Oliver, et al. 2011; Van Hooren and Zolotarjova 2017), whereas a comprehensive testing battery including a combination of both slow (CMJ) and fast (reactive strength index and leg stiffness) SSC actions could create a more accurate SSC profile of pre‐PHV children (R. S. Lloyd, Oliver, et al. 2011; McGarrigal et al. 2025b). Considering that developing the fast SSC has potential to increase athletic performance in sprinting (Chelly and Denis 2001; Rumpf et al. 2013; M. J. Taylor and Beneke 2012), reduce running economy (Arampatzis et al. 2006; Dalleau et al. 1998), increase jump height (Arampatzis et al. 2006, 2001) and reduce the risk of anterior cruciate ligament (ACL) injury (Brazier et al. 2014; Butler et al. 2003; Chimera et al. 2004; Hewett et al. 1996), strength and conditioning practitioners working with pre‐PHV children should tailor PT programmes aimed at developing both the slow (e.g., CMJ height) and fast (e.g., reactive strength index and leg stiffness) SSC (R. S. Lloyd, Oliver, et al. 2012; McGarrigal et al. 2025b). Using PT to reduce ACL injuries is especially important to practitioners working with youth female athletes (Chimera et al. 2004; McGarrigal et al. 2025a), as this population sustains more ACL injuries compared to boys playing the same sports (Beech et al. 2022; Bram et al. 2021; Childers et al. 2025; Robles‐Palazon et al. 2022; Sanchez‐Sanchez et al. 2025).

It should be acknowledged that this study had several limitations. For example, this study used PHV as a proxy for biological maturity, which is commonly used in sporting contexts because it relies on standard anthropometric measurements that are easily and routinely collected by practitioners (Asadi et al. 2017; Davies et al. 2019; R. S. Lloyd et al. 2014; Malina, Coelho‐e‐Silva, et al. 2021; Malina, Martinho, et al. 2021; McGarrigal et al. 2025b; Sylvester et al. 2024; K. Talukdar et al. 2021; T. Talukdar et al. 2021). Although more accurate methods, such as skeletal age assessment or clinical evaluation using Tanner stages, are considered the ‘gold standard’ (Malina et al. 2004b), they are less accessible outside a clinical setting (R. S. Lloyd et al. 2014). For instance, skeletal age assessment requires portable X‐ray equipment and trained personnel to obtain and interpret wrist radiographs, whereas Tanner staging requires a qualified health professional to conduct potentially sensitive physical examinations of secondary sexual characteristics (Malina et al. 2004b). The latter method, particularly the assessment of breast and pubic hair development, may cause discomfort or distress among adolescent female athletes (Moon and Davies 2023). Nevertheless, future research studies involving youth female soccer players may consider conducting assessments of skeletal age or Tanner staging, when feasible and ethically appropriate, with the support of qualified health professionals (R. S. Lloyd et al. 2014).

The PT programme included 2 sessions per week over a 6‐week period, which is popular frequency time frame undertaken by similar studies (Chimera et al. 2004; Miller et al. 2006; Ramirez‐Campillo, Gallardo, et al. 2015; Thomas et al. 2009). This was advantageous as this duration fits into an elementary school half term without the need of week's break with the PT programme (Moran et al. 2016). Additionally, one meta‐analysis (Slimani et al. 2017) found no significant difference between increases in CMJ height in male and female soccer players during PT lasting < 8 weeks and ≥ 8 weeks (*p* = 0.945). In the current study, the total number of foot contacts (1320) closely mirrored those of similar studies (McGarrigal et al. 2025b; Ramirez‐Campillo, Gallardo, et al. 2015; Türkarslan and Deliceoglu 2024). It is also possible, however, that a longer duration, frequency and time dedicated to perform PT could have increased the benefits of the PT observed in the current study (Moran et al. 2018). Specifically, in their meta‐analysis, Moran et al. 2018 reported a greater effect size and a significant difference in vertical jump height in PT > 8 weeks compared to a programme ≤ 8 weeks in girls aged 8–18 years (*p* = 0.004; ES = 1.04 vs. 0.24). The same authors also reported a greater effect size when performing > 2 sessions compared to ≤ 2 sessions (ES = 0.37 vs. 1.22) and PT performed for greater than 30 min compared to PT of less than 30 min (Moran et al. 2018). Therefore, future studies may wish to compare PT duration, frequency and volume in pre‐PHV children to determine optimal programing strategies across different stages of maturation. Overall, the findings reinforce that training adaptations in youth are multifactorial and influenced by both biological maturation and programme design, underscoring the need for individualized approaches rather than rigid age‐based prescriptions.

## 5 Conclusion

This study demonstrates that a 6‐week PT programme can positively influence CMJ height. Although the PG significantly outperformed the CG significantly in CMJ height following PT, only the PG girls significantly improved CMJ performance. Therefore, these findings suggest that PT can be performed in an elementary school setting to assist improvements in vertical jump performance of both sexes, but strength and conditioning practitioners may observe greater adaptations in 9–11‐year‐old girls compared to age‐match boys if the girls are closer to their PHV. Due to the principle of training specificity and its role in sports development, strength and conditioning practitioners working with children looking to improve vertical jump height should perform plyometric drills in vertical plane. Finally, as only take‐off velocity improved following PT, strength and conditioning practitioners looking to increase force and power should supplement PT with resistance‐based strength training in pre‐PHV children to better facilitate greater improvements in jump height performance and athletic performance.

## Conflicts of Interest

The authors declare no conflicts of interest.

## Data Availability

The data that support the findings of this study are available on request from the corresponding author. The data are not publicly available due to privacy or ethical restrictions.
